# Antecedents of patient health engagement in the radiotherapy service (evidence from Indonesia)

**DOI:** 10.4102/hsag.v28i0.2245

**Published:** 2023-12-13

**Authors:** Anthony Kasena, Ferdi Antonio

**Affiliations:** 1 Department of Hospital Administration, Faculty of Medicine, Universitas Pelita Harapan, Daerah Khusus Ibukota Jakarta, Indonesia

**Keywords:** patient health engagement, patient experience, revisit intention, intent to recommend, radiotherapy

## Abstract

**Background:**

Private radiotherapy (RT) facilities in emerging countries are growing with cancer incidence. Private healthcare providers must provide better care based on patient perspectives to reach more patients.

**Aim:**

This study investigated the relationship between antecedents of patient health engagement (PHE) with revisit intention (RVI) and intent to recommend (ITR) in private RT facilities.

**Setting:**

The survey was conducted in a private hospital with a RT service in Central Java province, Indonesia.

**Methods:**

A quantitative, cross-sectional design with a purposive sampling method was used. Patient questionnaire survey modified from validated self-administered radiotherapy experience (RTEQ) and PHE questionnaire were used to collect data. Partial least squares-structural equation modelling was used to analyse the data.

**Results:**

In this study, 173 respondents consented to participate, which demonstrated that seven of the eight experience antecedents of PHE measured by reliable and valid RTEQ and were significantly related to PHE (*p*-value <0.05). At the same time, the degree of PHE has a significant relationship with RVI and ITR (*p*-value <0.05).

**Conclusion:**

Patient informational needs elements from the patient experience, followed by situational repose, were shown to have a prominent relation to PHE. The management of private RT facilities needs to focus more on these elements to encourage PHE to establish hospital performance.

**Contribution:**

The findings denote that six elements of RTEQ relate to PHE and further hospital outcomes. Hospital management could utilise this approach to improve the quality of care in RT facilities, specifically in private hospitals in emerging countries.

## Introduction

Developing countries have ample growth in healthcare facilities and have particular healthcare problems. Hospitals offer a wide range of services with excellent service to attract patients and grow their business. The cancer treatment industry is attractive as cancer cases are getting more widespread worldwide, particularly in emerging countries. Patients are looking for better services during treatment that offer convenient and empathic services. The hospital should improve the quality of care inpatient treatment by establishing the system (Donabedian 1988). This approach should become patient-centred, activating patient involvement in their treatment plan (Bombard et al. 2018). Healthcare quality of services should also be assessed from the patient’s perspective (Abbasi-Moghaddam et al. 2019). Patient engagement is an essential concept according to the patient-centred paradigm. This concept became popular in the last few decades because of its helpfulness in assessing the quality of care (Tobiano, Jerofke-Owen & Marshall 2021). However, there has not been much research on patient engagement in specific fields, such as radiotherapy (RT) services. This is necessary because cancer cases requiring RT are increasing. This study aims to identify the relationship between patient engagement and hospital growth. Thus, research on this topic can provide new insights into hospital management.

As an emerging country, Indonesia has had a high demand for RT facilities during the past decade (Octavianus & Gondhowiardjo 2022). Cancer has contributed as a leading cause of death and is a critical problem that causes low life expectancy in almost all countries (Bray et al. 2021). According to data from 2020, there were 68 858 new incidences of breast cancer, or 16.6% of Indonesia’s total of 396 914 new cancer cases. Over 23 000 deaths were reported during this time (Sung et al. 2021). Cancer patients have various treatment modalities, including RT, immunotherapy, chemotherapy, hormone therapy and surgery. Radiotherapy, as one of the cancer treatments, is an essential part of both curative and palliative cancer care (Yap et al. 2016). Radiotherapy is also one of the most widely used methods for cancer treatment because of its low cost and high effectiveness (Burnette & Weichselbaum 2013).

A typical curative RT treatment duration ranges from 3 weeks to 8 weeks (Chaput & Regnier 2021). Given the length of treatment, developing a good long-term relationship between the healthcare provider and patient is critical. The patient could develop commitment from trust raised in the relationship (Morgan & Hunt 1994). Healthcare providers have a long record of measuring patient satisfaction levels with their services mainly on a functional aspect, such as how the service is delivered, but less on the clinical outcome or technical aspects, which involve the physical and psychological state (Swain & Kar 2018). Measuring patient satisfaction has been debated in the literature for decades, with the mission described as complex and challenging (Collins & Nicolson 2002). Moreover, the measurement of satisfaction surveys could hardly be transferred to the quality of care measurement (Fenton 2012). Therefore, the interaction between patient and healthcare provider should be incorporated in the measurement. This approach was more favourable to be described with patient experience (Wolf et al. 2014, 2021).

The patient experience concept is widely acknowledged as a distinct dimension of healthcare quality. The patient is a one-of-a-kind human being. They are referred to as patients when suffering from a disease, but they remain the same unique individual they have always been (Oben 2020). Patient experience is defined as the sum of all interactions, the impact of organisational cultures, patient perceptions and the significance of considering experiences across the continuum of care (Wolf et al. 2014, 2021). Cancer patients suffering from chronic diseases face the possibility of a potentially incurable life-threatening illness, a condition that can cause physical and psychological distress. The current application of the Radiotherapy Experience Questionnaire (RTEQ) helps measure patient comfort and experiences (Olausson et al. 2017); however, it is rarely used to assess its applicability regarding hospital outcomes.

Patient engagement is a new concept in the last few decades and is defined as a complex and multidimensional experience that results from an individual’s cognitive, emotional and affective towards their health promotion (Graffigna et al. 2014). As stated by a study, patient health engagement (PHE) can help healthcare professionals and policymakers customise their interventions to provide the most appropriate care management for patients and change the course of a disease (Barello et al. 2021). A patient’s ability to synergise the different stages of subjective dimensions (think, feel and act) during a specific period may impact engagement. According to the PHE model’s process, there are several levels this model could describe; depending on their emotional, cognitive and behavioural perspectives, people may engage in care management differently (Graffigna et al. 2015). For instance, a patient who receives a critical diagnosis may be unable to manage care due to the emotional effect. Patient engagement is also described as an active, cooperative interaction between patients and researchers about treatment plans in which the patients participate as partners and make decisions while sharing particular experiences and service values (Harrington et al. 2020).

Additionally, the idea of patient engagement is consistent with the description of one study in which the definition is the willingness and capacity to actively choose to play an active role in the care that is particularly relevant to the individual, in collaboration with a healthcare practitioner or institutions, for the sake of achieving higher health outcomes or fostering experiences of care (Higgins, Larson & Schnall 2017). The healthcare system must offer more organised support and consider caregivers’ primary requirements and objectives. According to this current view, PHE may be preferable to assess the long-term relationship and commitment between patients and healthcare providers based on care delivery (Hahn et al. 2021).

Measuring the willingness to consider a recommendation to others thus can be viewed as an essential factor in assessing the company’s future performance (Purificacion et al. 2016). Given the highly competitive market for private RT facilities today, to gain more customers or patients, it was critical to implement a novel strategy to increase services and outperform the competition (Güçer & Arıcı 2018). Patient health engagement affected by the patient experience could impact the patient’s intention to patroness the hospital benefit, such as revisit intention (RVI) and intent to recommend (ITR), so it could increase consumers’ RVI by enhancing the quality of medical services (Park et al. 2021). Revisit intention and ITR should become an essential consideration for stakeholders because retaining the existing customer and improving compliance or completing the therapy schedule are essential (Yan, Wang & Chau 2015). Therefore, PHE can be used to predict patient intention. As a result, PHEs function as a mediation between patient experience and healthcare providers, and result in the organisational outcome.

Researchers noted the links between patient experience with conformity to shared care plans and patient engagement (Stults et al. 2016). In the RT setting, it was stated that situational unease, physical discomfort, situational repose, informational needs (INF), treatment environment acceptance and patient trust are essential in defining the RT patient experience (Olausson et al. 2017). Based on a recent study, patient experience may lead to adherence to shared improved patient engagement (Holt 2018). Therefore, the authors suggest the hypothesis:

- **H1:** Manageable situational unease has a positive relationship with patient health engagement.
- **H2:** Manageable physical discomfort has a positive relationship with patient health engagement.
- **H3:** Situational repose has a positive relationship with patient health engagement.
- **H4:** Informational needs have a positive relationship with patient health engagement.
- **H5:** Treatment environment acceptance has a positive relationship with patient health engagement.
- **H6:** Level of trust and understanding has a positive relationship with patient health engagement.

The quality of care dramatically influences patient well-being; the more significant the patient’s well-being, the higher the degree of engagement and the more the patient will support the healthcare provider (Lumentut & Antonio 2022). In healthcare, patient engagement is crucial for researchers to analyse the patient’s intentions. According to a recent study, PHE elements could be a precursor to the patient’s intention (Amin et al. 2022; Graffigna et al. 2020). To the best of our understanding, there has never been a study that concentrated on the connection between patient experience as measured by PHE and the intention to revisit and recommend variables, particularly in RT. Thus, the authors suggest the following hypotheses:

- **H7:** Patient health engagement has a positive relationship with revisit intention.
- **H8:** Patient health engagement has a positive relationship with intent to recommend.

**FIGURE 1:**
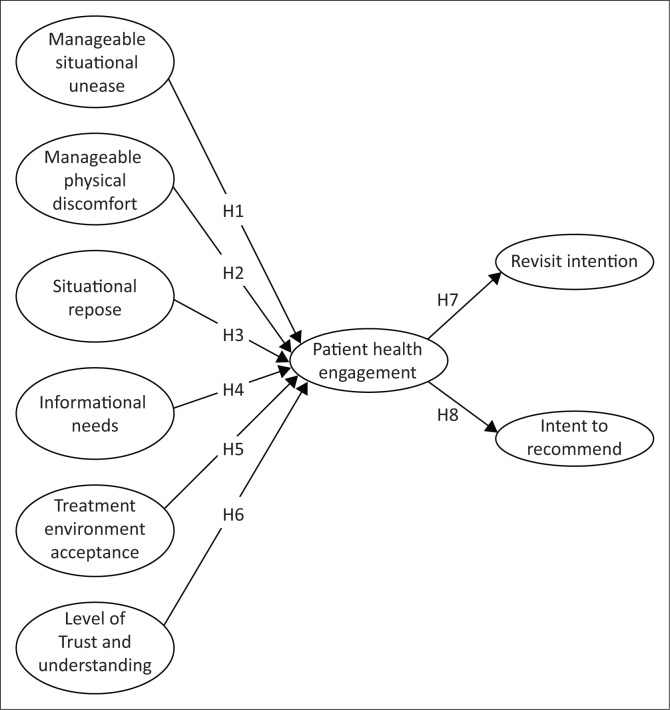
Conceptual framework.

This study proposes a new research framework (Figure 1) based on previous studies on hospital patient care in a more specific population, the cancer patient (Graffigna et al. 2020). This study investigated the relationship between antecedents of PHE with RVI and ITR in private RT facilities. In addition, the institution’s contribution is identifying the elements that increase the number of patients from RVI and ITR to improve service and provider growth. The dependent variables are RVI and ITR. At the same time, PHE in this study model has become a target construct that mediates patient experience to the RVI and ITR. Furthermore, the elements of RTEQ become six independent variables as antecedents of PHE.

## Methods

### Study design

In this study design, the authors conducted an analytical, cross-sectional quantitative study with a questionnaire gathered from participants at a specific period. Partial least squares-structural equation modelling (PLS-SEM) was used as the data analysis method to investigate the relationship between variables.

### Setting

This study occurred in a private hospital, RSKS, located in Central Java, Indonesia. This hospital has 10 years of experience and a complete infrastructure, two RT machines and one-stop oncology treatment. Only a few regional hospitals have these services. Thus, it has many patients and is becoming one of the referral hospitals. This hospital has been accredited five stars by the Indonesian Hospital Accreditation Commission and certified by the Indonesian Nuclear Energy Regulatory Agency (BAPPETEN).

### Study population and sampling strategy

The framework was tested with a purposive sampling technique on 216 participants obtained from a population of RT patients who underwent RT treatment in one private hospital in 2022. The participants were recruited with inclusive criteria: the patients who have received RT treatment more than three times and whose recent treatment was within the previous year. As for the exclusive criteria: unconscious patient who cannot communicate or answer the questionnaire and currently experiencing excruciating pain. A total of 216 responses were received 4 weeks after the questionnaire’s deployment at the outpatient RT department. After applying the eligibility criteria, 173 participants remained. The number of samples in this study was determined by power analysis using G-power with *f*^2^ 0.15, and the sample number required was 153 (Memon et al. 2020; Sarstedt et al. 2022). This statement aligns with recommendations for PLS-SEM; the minimum sample required was 160 participants (Kock & Hadaya 2018). Therefore, 173 participants in this study are qualified.

### Data collection

Data were gathered using a validated self-administered questionnaire. A collection of indicators measures the proposed conceptual framework’s constructs in a structured questionnaire instrument. This conceptual framework assessed six independent variables (MSU, manageable physical discomfort [MPD], SR, INF, TEA and LTU) to investigate how the antecedent of PHE affected the dependent variables (RVI and ITR). The questionnaire used in this study was created from a prior study and modified to meet the inquiry requirements. On a scale of 1–6, participants were asked to rate how much they agreed with the claims in the questionnaire, ranging from 1 (strongly disagree) to 6 (strongly agree). The RTEQ questionnaire was adopted from Olausson et al. (2017). As for the PHE questionnaire, several levels of engagement were measured depending on patients’ emotional, cognitive and perspective along their treatment journey (Graffigna et al. 2015). The RVI and ITR questionnaires were adapted from Octavius and Antonio (2021) and Park et al. (2021). In order to ensure that all of the components are clearly understood, these questionnaires have already been translated into the local tongue. A university language expert reviewed this questionnaire in the field of healthcare research before it was issued. The assigned healthcare professionals delivered the questionnaire in the RT outpatient department. Participants in the questionnaire voluntarily consented to participating anonymously and without disclosing their identities after being given assurances that the data they provided would be kept confidential. Informed consent was applied in the first section of the questionnaire, and no incentives were given. The data were collected from August 2022 to September 2022.

### Data analysis

This study used the PLS-SEM method because it could analyse complex models in explanatory research. The conceptual framework comprises nine constructs and is considered a complex research model. When a study focuses mainly on the model’s explanatory power, PLS-SEM approaches are used (Hair et al. 2019). SmartPLS version 3.2.9 was used to conduct the PLS-SEM analysis, as it has a bootstrapping option to determine significance (Memon et al. 2020). Two different models, namely measurement and structural models, are the foundation of the PLS-SEM primary procedure. The measuring model was developed to assess the reliability and validity of the model’s components and indicators. The testing phase includes indicator reliability (outer loading) and construct reliability (Cronbach’s alpha [CA]) and composite reliability (CR). Construct validity (average variance extracted [AVE]) and discriminant validity are included in the validity testing process (heterotrait-monotrait [HT/MT] ratio). The process can move on to the following stage if all four items are reliable and valid. The structural model analyses how important it is that certain constructs in the research model are related to one another. A mediation analysis will also be performed to evaluate the elements in this model that act as mediators. This work uses importance-performance map analysis (IPMA), a more advanced PLS technique, for particular management implications (Ringle & Sarstedt 2016).

### Ethical consideration

The RSKS Research and Ethics Committee has examined the research protocol, including the information provided to the potential subjects, entitled ‘Antecedents of Patient Health Engagement in the Radiotherapy Service (Evidence from Indonesia)’, ensuring welfare and human rights in study (ref. no. 370/EL/RSKS/2022). There were no risks associated with the study.

## Results

### Demographic results

In the chosen RT facility, 216 total responses were recorded. After applying inclusion criteria, there remained 173 valid responses from patients who received RT treatment more than three times and whose most recent treatment was within the previous year, as described in Table 1. Most respondents (75.7%) received RT treatment more than six times and visited the facility for less than a month (62.4%). Most patients are female (67.1%) and between the ages of 45 years and 65 years make up the majority (69.5%) of the patient population. About 89.6% of the patient’s origins are in Central Java. Most respondents (46.2%) had junior high school as their most recent educational level, and 44.5% were housewives. The patient could generally complete daily tasks without assistance (80.9%) and took no painkillers in their treatment (67.1%). Most responders are independent and can care for themselves, and survey participants can converse and respond to the questions. All patients (100%) had their payment method covered by national health insurance Badan Penyelenggara Jaminan Social (BPJS), and for the majority of patients (51.4%), the cancer diagnosis has been recognised for longer than a year.

**TABLE 1:** Respondents profile.

Demographic variable	Sample (*n*)	%
**Last time visit to RT facility for treatment**		
< 1 month	108	62.4
1–6 months	44	25.4
6–12 months	21	12.1
**RT treatment performed in the last 12 months**		
3–6 times	42	24.3
> 6 times	131	75.7
**Age**		
18–24 years	8	4.6
25–44 years	45	26.0
45–65 years	120	69.4
**Gender**		
Male	57	32.9
Female	116	67.1
Choose not to answer	0	-
**Domicile**		
Central Java	155	89.6
West Java	0	-
East Java	18	10.4
Others	0	-
**Last education**		
Junior high school	81	46.8
Senior high school	61	35.3
Bachelor degree (undergraduate)	27	15.6
Master’s degree (postgraduate)	4	2.3
**Occupation**		
Student	5	2.9
Self-employed	28	16.2
Government employees	9	5.2
Employee	37	21.4
Housewife	77	44.5
Freelance	3	1.7
Others	14	8.1
**Pain killer consumption**		
Yes	57	32.9
No	116	67.1
**Ability to perform daily activities without help**		
Yes	140	80.9
No	33	19.1
**Duration of cancer has been diagnosed**		
< 6 months	28	16.2
6–12 months	56	32.4
> 12 months	89	51.4
**Payment method**		
Self	0	-
National health insurance (BPJS)	173	100.0
Other insurances	0	-

RT, radiotherapy.

### Measurement model

In the first step, the outer loading value is analysed, and the reliability of the reflective indicator is assessed. As shown in Table 2, all 30 research variable indicators met the outer loading criterion, demonstrating the reliability of every indicator used in this study (>0.708) (Hair et al. 2019; Sarstedt et al. 2022). The next step is to analyse the construct reliability to verify the model’s internal consistency and avoid redundancy. Each variable value has a Cronbach’s alpha greater than 0.7 and a composite reliability between 0.7 and 0.95, indicating reliability (Hair et al. 2019, 2022). The AVE is assessed to determine convergence validity. The AVE is assessed to determine convergence validity (AVE). All indicators are reliable as the AVE score exceeds 0.50, indicating that the constructs can sufficiently represent at least 50% of the variance (Hair et al. 2019, 2022).

**TABLE 2:** Reliability and validity analysis.

Variables	Indicators		Outer loadings	CA	CR	AVE
MSU	MSU1	I rarely get frustrated with radiotherapy.	0.742	0.826	0.898	0.747
	MSU2	I rarely feel afraid during radiotherapy.	0.929			
	MSU3	I rarely feel anxious during radiotherapy.	0.909			
MPD	MPD1	I rarely feel pain when the nurse adjusts my position.	0.873	0.789	0.874	0.697
	MPD2	I rarely feel pain when I lie on the radiotherapy table.	0.825			
	MPD3	I rarely feel pain during radiotherapy procedures.	0.806			
SR	SR1	I often feel calm during radiotherapy.	0.869	0.832	0.850	0.751
	SR2	I feel safe during radiotherapy (from the effects of radiation).	0.933			
	SR3	I am comfortable while in the radiotherapy room.	0.791			
INF	INF1	I have enough information about the side effects of radiotherapy.	0.886	0.904	0.933	0.778
	INF2	I have enough information about how to deal with radiotherapy side effects.	0.921			
	INF3	I have enough information about the benefits of radiotherapy that I am undergoing.	0.875			
	INF4	I am well-informed about how radiotherapy works.	0.844			
TEA	TEA1	For me, the vibe of this radiotherapy room gives a comfortable impression.	0.714	0.740	0.853	0.662
	TEA2	For me, the lighting of this radiotherapy room is quite comfortable (bright enough and not glare).	0.828			
	TEA3	For me, this radiotherapy room looks clean.	0.888			
LTU	LTU1	I feel trust in the radiotherapy personnel.	0.893	0.912	0.944	0.850
	LTU2	I understand the radiotherapy procedure that I am undergoing.	0.934			
	LTU3	I believe in the professional attitude shown by the radiotherapist.	0.938			
PHE	PHE1	I seem to want to lie down or blackout.	0.826	0.923	0.942	0.764
		I feel restless.				
		Neutral				
		I’m fully aware.				
		I feel optimistic.				
	PHE2	I feel confused.	0.854			
		I feel suffering.				
		Neutral				
		I can understand my condition.				
		I feel calm.				
	PHE3	When I think of my illness, I feel sad.	0.902			
		I feel anxious when new symptoms appear.				
		Neutral				
		I feel used to my disease condition.				
		Despite the illness I’m experiencing, I feel like I can still live life.				
	PHE4	I feel hopeless because of my illness.	0.890			
		I often feel anxious during the treatment process.				
		Neutral				
		I feel I can adjust to my illness.				
		I am generally optimistic about the future as well as my health condition.				
	PHE5	I feel oppressed by my illness.	0.897			
		I am upset when new symptoms arises.				
		Neutral				
		I feel I have accepted my illness.				
		I can give sense to my life despite my illness condition.				
RVI	RVI1	I plan to revisit this hospital shortly.	0.910	0.850	0.909	0.770
	RVI2	If I need treatment, I will choose to return to this hospital.	0.888			
	RVI3	I choose this hospital compared to other radiotherapy hospitals if I need treatment.	0.832			
ITR	ITR1	I will recommend this facility to those who require it.	0.830	0.820	0.892	0.733
	ITR2	I am pleased to inform people that this hospital’s radiotherapy is pleasant.	0.845			
	ITR3	I want to share positive things about radiotherapy at this hospital.	0.893			

AVE, average variance extracted; CA, Cronbach’s alpha; CR, composite reliability; MSU, manageable situational unease; MPD, manageable physical discomfort; SR, situational repose; INF, informational needs; TEA, treatment environment acceptance; LTU, level of trust and understanding; PHE, patient health engagement; RVI, revisit intention; ITR, intent to recommend.

The HT/MT ratio is evaluated by observing the outer model using the discriminant validity test. To clearly distinguish the indicator’s construct, it is advised that the HT/MT ratio is set at a value of 0.9 (Henseler, Ringle & Sarstedt 2015; Sarstedt et al. 2022). Table 3 presents the results and identifies each indicator measuring a particular construct. The structural model can proceed to the next stage based on the findings of the outer proposed model, which show that all of the indicators in the research framework are reliable and valid.

**TABLE 3:** Discriminant validity with heterotrait-monotrait ratio.

Variable	INF	ITR	LTU	MPD	MSU	PHE	RVI	SR	TEA
INF	-	-	-	-	-	-	-	-	-
ITR	0.614	-	-	-	-	-	-	-	-
LTU	0.771	0.610	-	-	-	-	-	-	-
MPD	0.792	0.832	0.724	-	-	-	-	-	-
MSU	0.529	0.729	0.642	0.661	-	-	-	-	-
PHE	0.717	0.617	0.709	0.634	0.632	-	-	-	-
RVI	0.597	0.761	0.639	0.621	0.620	0.670	-	-	-
SR	0.642	0.755	0.729	0.759	0.727	0.702	0.688	-	-
TEA	0.697	0.736	0.710	0.797	0.742	0.728	0.620	0.786	-

MSU, manageable situational unease; MPD, manageable physical discomfort; SR, situational repose; INF, informational needs; TEA, treatment environment acceptance; LTU, level of trust and understanding; PHE, patient health engagement; RVI, revisit intention; ITR, intent to recommend.

### Structural model

The second stage of analysis, known as the inner model analysis, was carried out to determine how well the model used in this study predicted the relationship between the variables. The variance inflation factor (VIF) was used to check common method bias and multicollinearity, while *R*-square (*R*^2^) and *Q*-square (*Q*^2^) were used to assess the prediction capability of the model. This multicollinearity test did not reveal collinearity problems because all VIF values were below 5, as suggested by Hair et al. (2019, 2022). As can be seen in Figure 3, the PHE *R*^2^ value results (0.576) have a moderate explanatory power for values >0.5 (Hair et al. 2019, 2022). The PLS prediction procedure was employed to determine the proposed model’s out-of-sample predictive value, which was then applied to assess the suggested model’s predictive potency. The PHE *Q*^2^ (0.544) construct predicts values that were found to be pertinent and demonstrate significant predictive relevance (> 0.50), indicating that this research model has the necessary capacity to predict PHE in the various samples. Revisit intention and ITR explanatory power are demonstrated by their respective *R*^2^ values of 0.353 and 0.303. The construct can predict values with moderate predictive relevance, as shown by the RVI and ITR *Q*^2^ findings of 0.369 and 0.392, respectively (Hair et al. 2019; Shmueli et al. 2019).

The significance of variables was assessed using the bootstrapping protocol, and all of the model’s hypotheses were confirmed. *P*-value <0.05 and a *T*-statistic >1.645 (one-tailed with 0.05) with a confidence interval (CI) between the range of 5% and 95% to assess the significance of the hypothesis (Hair et al. 2022). Seven hypotheses were supported with a *T*-statistic >1.645, *p*-value <0.05, and 95% CI with positive values. Except for H3, the standardised coefficient was positive, consistent with how the hypotheses evolved (Table 4). The INF standard coefficient value (H4), an antecedent of PHE, was the highest (0.314). According to the standardised coefficient of PHE to RVI in private RT services, PHE is found to be predominantly related to RVI (0.594).

**TABLE 4:** Significance and coefficient.

Variable	Hypothesis	Std. deviation	*T*-statistics	*P*	CI 5.0%	CI 95.0%	Results
H1	Manageable situational unease -> Patient health engagement	0.144	2.578	0.005	0.052	0.236	Hypothesis supported
H2	Manageable physical discomfort -> Patient health engagement	−0.052	0.687	0.246	−0.173	0.081	Hypothesis not supported
H3	Situational repose -> Patient health engagement	0.177	3.029	0.001	0.084	0.276	Hypothesis supported
H4	Informational needs -> Patient health engagement	0.314	5.221	0.000	0.208	0.404	Hypothesis supported
H5	Treatment environment acceptance -> Patient health engagement	0.159	2.621	0.004	0.069	0.268	Hypothesis supported
H6	Level of trust and understanding -> Patient health engagement	0.175	2.834	0.002	0.073	0.277	Hypothesis supported
H7	Patient health engagement -> Revisit intention	0.594	11.646	0.000	0.516	0.684	Hypothesis supported
H8	Patient health engagement -> Intent to recommend	0.551	10.489	0.000	0.468	0.640	Hypothesis supported

Std, Standard; CI, confidence interval; H, hypotheses.

In addition, mediation analysis was performed following Nitzl, Roldan and Cepeda (2016) to ascertain the mediation’s importance through the specific indirect effects. Based on the findings of the mediation study, the supported hypotheses had *T*-statistics and *p*-value < 0.05 for PHE, and all the mediator constructs investigated were above the 1.645 criteria except for the MPD path. This outcome demonstrates that PHE is a significant mediator from the antecedents of PHE to RVI and ITR.

**FIGURE 2:**
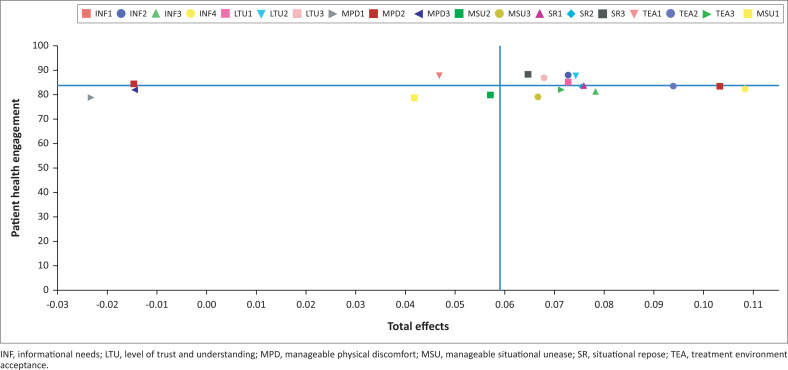
Importance-performance map of indicators.

Importance-performance map analysis was used to identify the areas that need to be improved in RT services. Figure 2 shows which indicators should be maintained or enhanced in each quadrant based on importance (total effect) and performance (Ringle & Sarstedt 2016).

INF4, INF1, INF2 and INF3 (by total effect order) were all found in quadrant IV of Figure 2 (right lower side). Given that they were important for patients but have yet to be performed adequately, this finding strongly suggests that RT facility management should pay more for these indicators, primarily the INF indicators.

## Discussion

**FIGURE 3:**
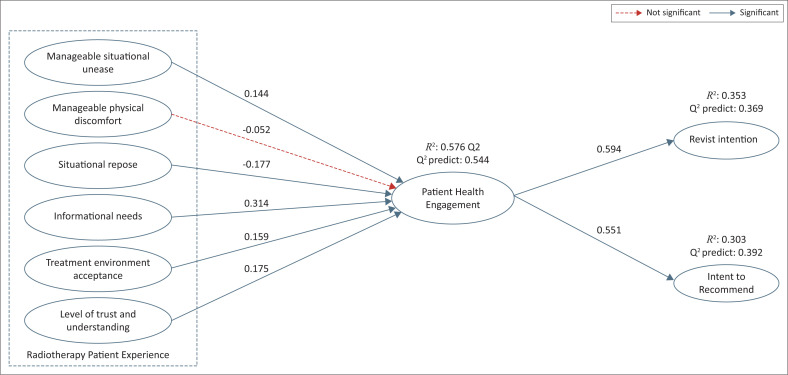
Empirical model.

Given the need for private healthcare providers in developing countries to compete in the market, this study emphasises the concept of patient-centred care from their perspective. The study’s findings (Figure 3), following the earlier empirical studies (Graffigna et al. 2020) and its addition demonstrate that concerning private RT service facilities, the increases of PHE have been associated with higher RVI and ITR. This empirical research was done particularly on cancer patients, while the earlier study of PHE rarely included it, thus leading to new insight from a cancer patient perspective.

This study’s result aligns with the previous study (Holt 2018). Thus, patient experience is essential for evaluating healthcare quality reflected by PHE. Therefore, it could be suggested to the hospital manager to consider the feedback based on the patient report on its experience to improve the service. The five PHE antecedents in this study (RT patient experience) were shown in Figure 3 to be significantly associated, except for MPD. This insignificant result may be related to the subjective evaluation, where people’s perceived pain varies from person to person and is influenced by their unique physiological, emotional and cognitive states (Wilcox et al. 2015). Regarding this study, 33% of samples use strong painkillers in addition to their treatment. While the rest, 67% of the participants, used painkillers occasionally, which means they could manage the pain.

The standardised coefficient value of INF to PHE was higher than other independent variables (0.314). Patient health engagement increases with the information obtained following their needs (Mirzaei and Esmaeilzadeh 2021). This finding was in line with a recent study that shows that it is crucial to address their INF to increase patient participation (Eriksson-Liebon, Roos & Hellström 2021). Positive PHE with the information provided is one crucial measure of the quality of care provided; the well-informed patient is an essential aspect that should be the primary focus of healthcare practitioners (Davis, Schoenbaum & Audet 2005). Therefore, assessing the patient’s information needs at each RT session is advised. Radiation oncologists should also ensure patients understand and remember the information they give during the session (Douma et al. 2012). There is a consensus that using different information methods, such as verbal, written and so forth, has advantages in provider and patient interaction (Azad et al. 2021). This study’s results support the notion that healthcare professionals will eventually need to use a good mix of ways to satiate each patient’s information needs and preferences to boost engagement (Durnin, Shepherd & Gilleece 2021). The INF should be given priority by management when allocating time and resources as it is believed to be an essential component for patients and needs to improve if it has not performed as expected (Figure 2).

The degree of patient engagement significantly impacts cancer patients’ perception of the care received. Patients recently diagnosed typically engage less and struggle to accept their condition. However, patients receiving treatment and having a long diagnosis history are more engaged. Healthcare professionals must be aware of this to deliver patients the required services. The PHE scale in this study can identify the complex and subtle psychological well-being of PHE and has an ordinal structure to be aligned with the concepts of the PHE model. The research findings show that all PHE indicators are valid and reliable, as shown in Table 2. The importance of these indicators is that they could describe the patients on several levels. They have a ‘low PHE score’ in the blackout or alert situation. However, when they had a ‘high PHE score’, their scoring was associated with adhesion and eudemonic orientation (Graffigna et al. 2015). This study denotes that patients’ level of engagement is significantly associated with how they evaluate the care or service they receive, which is related to their future behaviour; therefore, it is essential to assess patient engagement to improve the quality of care.

This study’s findings are consistent with research done by Graffigna et al. (2020) and Amin et al. (2022), and it can be deduced that PHE could be used as an element when determining ITR and RVI in a private hospital. This outcome of patient evaluations may also impact the financial performance of medical facilities. The proposed model shows that *R*^2^ values for RVI and ITR have moderate explanatory power. In contrast, the *Q*^2^ value shows that the model is still predicted when the parameters are altered, thus indicating a susceptible model.

A new contribution of this study to the health management literature is to demonstrate a novel approach that shows elements of RT patient experience has a significant relation with PHE. The outcome of patient evaluations is significantly associated with their RVI and ITR, both of which could lead to the business performance of the medical facilities. Aside from measuring patient satisfaction, healthcare management and practitioners are suggested to be more aware of patients’ health engagement levels and the benefit of patient support to the hospital reflected by patronised intention. Given that it is beneficial for predicting RVI and ITR in private RT facilities, future studies might extend and test this model on a greater number of demographic statistics.

## Limitations and recommendations

Some of the study’s limitations should be considered in subsequent research on related topics. The study’s primary sample source was conducted on a single private RT facility. As for the recommendation, recruiting participants from various RT facilities across the country may be advised to obtain more accurate results in future research and a more representative sample with objective criteria.

The study participants did not classify the respondent based on cancer stage or diagnosis. Patients newly diagnosed and those receiving therapy for a while may have different levels of patient engagement with their health. Therefore, the recommendation is that staging, diagnosis, patient complaints and quality of life are better classified in future studies.

Lastly, because the patient treatment experience is the main focus, this study should have mentioned who was in direct contact with patients (doctors, nurses, radiographers) or who gave direct information to the patient. To fulfil patient INF, the care provider should be appointed, and the system should mention who is responsible for providing patient information to comprehend the information better.

## Conclusion

This study concludes that PHE positively correlates with RVI and ITR in the RT setting. The significant elements of RT patient experience are manageable situational unease, situational repose, INF, treatment environment acceptance, and level of trust and understanding measured by the RTEQ questionnaire, which is significantly proved as an antecedent of PHE. The proposed model shows how patients appraise services will depend on their perceptions and experiences of using private RT providers’ services. Higher levels of intention that benefit the hospital are present in patients who are positively engaged in their therapeutic process. To increase RVI and ITR, RT management must focus on PHE and carefully monitor the PHE indicators. Finally, patient needs for better information should also be considered to deliver better care quality in private hospitals.

## References

[CIT0001] Abbasi-Moghaddam, M.A., Zarei, E., Bagherzadeh, R., Dargahi, H. & Farrokhi, P., 2019, ‘Evaluation of service quality from patients’ viewpoint’, *BMC Health Services Research* 19(1), 170. 10.1186/s12913-019-3998-0 30876453 PMC6420766

[CIT0002] Amin, R., Hossain, M.A., Uddin, M.M., Jony, M.T.I. & Kim, M., 2022, ‘Stimuli influencing engagement, satisfaction, and intention to use telemedicine services: An integrative model’, *Healthcare (Basel, Switzerland)* 10(7), 1327. 10.3390/healthcare10071327 35885854 PMC9318589

[CIT0003] Azad, A., Sernbo, E., Svärd, V., Holmlund, L. & Björk Brämberg, E., 2021, ‘Conducting in-depth interviews via mobile phone with persons with common mental disorders and multimorbidity: The challenges and advantages as experienced by participants and researchers’, *International Journal of Environmental Research and Public Health* 18(22), 11828. 10.3390/ijerph182211828 34831582 PMC8619936

[CIT0004] Barello, S., Guida, E., Leone, S., Previtali, E. & Graffigna, G., 2021, ‘Does patient engagement affect IBD patients’ health-related quality of life? Findings from a cross-sectional study among people with inflammatory bowel diseases’, *Health and Quality of Life Outcomes* 19(1), 77. 10.1186/s12955-021-01724-w 33678181 PMC7938585

[CIT0005] Bombard, Y., Baker, G.R., Orlando, E., Fancott, C., Bhatia, P., Casalino, S. et al., 2018, ‘Engaging patients to improve quality of care: A systematic review’, *Implementation Science* 13(1), 98. 10.1186/s13012-018-0784-z 30045735 PMC6060529

[CIT0006] Bray, F., Laversanne, M., Weiderpass, E. & Soerjomataram, I., 2021, ‘The ever-increasing importance of cancer as a leading cause of premature death worldwide’, *Cancer* 127(16), 3029–3030. 10.1002/cncr.33587 34086348

[CIT0007] Burnette, B. & Weichselbaum, R.R., 2013, ‘Radiation as an immune modulator’, *Seminars in Radiation Oncology* 23(4), 273–280. 10.1016/j.semradonc.2013.05.009 24012341

[CIT0008] Chaput, G. & Regnier, L., 2021, ‘Radiotherapy: Clinical pearls for primary care’, *Canadian Family Physician Medecin De Famille Canadien* 67(10), 753–757. 10.46747/cfp.6710753 34649900 PMC8516179

[CIT0009] Collins, K. & Nicolson, P., 2002, ‘The meaning of “satisfaction” for people with dermatological problems: Reassessing approaches to qualitative health psychology research’, *Journal of Health Psychology* 7(5), 615–629. 10.1177/1359105302007005681 22113145

[CIT0010] Davis, K., Schoenbaum, S.C. & Audet, A.-M., 2005, ‘A 2020 vision of patient-centered primary care’, *Journal of General Internal Medicine* 20(10), 953–957. 10.1111/j.1525-1497.2005.0178.x 16191145 PMC1490238

[CIT0011] Donabedian, A., 1988, ‘The quality of care. How can it be assessed?’, *JAMA: The Journal of the American Medical Association* 260(12), 1743–1748. 10.1001/jama.260.12.1743 3045356

[CIT0012] Douma, K.F.L., Koning, C.C.E., Zandbelt, L.C., De Haes, H.C.J.M. & Smets, E.M.A., 2012, ‘Do patients’ information needs decrease over the course of radiotherapy?’, *Supportive Care in Cancer* 20(9), 2167–2176. 10.1007/s00520-011-1328-0 22081119 PMC3411284

[CIT0013] Durnin, R., Shepherd, P. & Gilleece, T., 2021, ‘An evaluation of the information needs of radiotherapy patients and their families’, *Journal of Radiotherapy in Practice* 20(4), 473–479. 10.1017/S1460396920000497

[CIT0014] Eriksson-Liebon, M., Roos, S. & Hellström, I., 2021, ‘Patients’ expectations and experiences of being involved in their own care in the emergency department: A qualitative interview study’, *Journal of Clinical Nursing* 30(13–14), 1942–1952. 10.1111/jocn.15746 33829575

[CIT0015] Fenton, J.J., 2012, ‘The cost of satisfaction: A national study of patient satisfaction, health care utilization, expenditures, and mortality’, *Archives of Internal Medicine* 172(5), 405. 10.1001/archinternmed.2011.1662 22331982

[CIT0016] Graffigna, G., Barello, S., Bonanomi, A. & Lozza, E., 2015, ‘Measuring patient engagement: Development and psychometric properties of the Patient Health Engagement (PHE) Scale’, *Frontiers in Psychology* 6, 274. 10.3389/fpsyg.2015.00274 25870566 PMC4376060

[CIT0017] Graffigna, G., Barello, S., Libreri, C. & Bosio, C.A., 2014, ‘How to engage type-2 diabetic patients in their own health management: Implications for clinical practice’, *BMC Public Health* 14, 648. 10.1186/1471-2458-14-648 24966036 PMC4083034

[CIT0018] Graffigna, G., Palamenghi, L., Boccia, S. & Barello, S., 2020, ‘Relationship between citizens’ health engagement and intention to take the COVID-19 vaccine in Italy: A mediation analysis’, *Vaccines* 8(4), 576. 10.3390/vaccines8040576 33019663 PMC7711984

[CIT0019] Güçer, E. & Arıcı, N.Ç., 2018, ‘The antecedents of revisit intention in medical businesses’, *Journal of Business Research – Turk* 10, 740–757. 10.20491/isarder.2018.453

[CIT0020] Hahn, E.E., Baecker, A., Shen, E., Haupt, E.C., Wakach, W., Ahuja, A. et al., 2021, ‘A patient portal-based commitment device to improve adherence with screening for colorectal cancer: A retrospective observational study’, *Journal of General Internal Medicine* 36(4), 952–960. 10.1007/s11606-020-06392-y 33474640 PMC8042087

[CIT0021] Hair, J.F., Hult, G.T.M., Ringle, C.M. & Sarstedt, M., 2022, *A primer on partial least squares structural equation modelling (PLS-SEM)*, 3rd edn., Sage, Los Angeles, CA.

[CIT0022] Hair, J.F., Risher, J.J., Sarstedt, M. & Ringle, C.M., 2019, ‘When to use and how to report the results of PLS-SEM’, *European Business Review* 31(1), 2–24. 10.1108/EBR-11-2018-0203

[CIT0023] Harrington, R.L., Hanna, M.L., Oehrlein, E.M., Camp, R., Wheeler, R., Cooblall, C. et al., 2020, ‘Defining patient engagement in research: Results of a systematic review and analysis: Report of the ISPOR Patient-Centered Special Interest Group’, *Value in Health: The Journal of the International Society for Pharmacoeconomics and Outcomes Research* 23(6), 677–688. 10.1016/j.jval.2020.01.019 32540224

[CIT0024] Henseler, J., Ringle, C.M. & Sarstedt, M., 2015, ‘A new criterion for assessing discriminant validity in variance-based structural equation modelling’, *Journal of the Academy of Marketing Science* 43(1), 115–135. 10.1007/s11747-014-0403-8

[CIT0025] Higgins, T., Larson, E. & Schnall, R., 2017, ‘Unravelling the meaning of patient engagement: A concept analysis’, *Patient Education and Counseling* 100(1), 30–36. 10.1016/j.pec.2016.09.002 27665500

[CIT0026] Holt, J.M., 2018, ‘An evolutionary view of patient experience in primary care: A concept analysis’, *Nursing Forum* 53(4), 555–566. 10.1111/nuf.12286 30196531

[CIT0027] Kock, N. & Hadaya, P., 2018, ‘Minimum sample size estimation in PLS-SEM: The inverse square root and gamma-exponential methods: Sample size in PLS-based SEM’, *Information Systems Journal* 28(1), 227–261. 10.1111/isj.12131

[CIT0028] Lumentut, I.J. & Antonio, F., 2022, ‘Quality of care effect on cancer patient’s well-being and its impact on the private hospital reputation’, *Universal Journal of Public Health* 10(5), 492–504. 10.13189/ujph.2022.100507

[CIT0029] Memon, M.A., Ting, H., Cheah, J.-H., Thurasamy, R., Chuah, F. & Cham, T.H., 2020, ‘Sample size for survey research: Review and recommendations’, *Journal of Applied Structural Equation Modeling* 4(2), i–xx. 10.47263/JASEM.4(2)01

[CIT0030] Mirzaei, T. & Esmaeilzadeh, P., 2021, ‘Engagement in online health communities: Channel expansion and social exchanges’, *Information & Management* 58(1), 103404. 10.1016/j.im.2020.103404

[CIT0031] Morgan, R.M. & Hunt, S.D., 1994, ‘The Commitment-Trust Theory of Relationship Marketing’, *Journal of Marketing* 58(3), 20–38. 10.1177/002224299405800302

[CIT0032] Nitzl, C., Roldan, J.L. & Cepeda, G., 2016, ‘Mediation analysis in partial least squares path modelling: Helping researchers discuss more sophisticated models’, *Industrial Management & Data Systems* 116(9), 1849–1864. 10.1108/IMDS-07-2015-0302

[CIT0033] Oben, P., 2020, ‘Understanding the patient experience: A conceptual framework’, *Journal of Patient Experience* 7(6), 906–910. 10.1177/2374373520951672 33457518 PMC7786717

[CIT0034] Octavianus, S. & Gondhowiardjo, S., 2022, ‘Radiation therapy in Indonesia: Estimating demand as part of a National Cancer Control Strategy’, *Applied Radiation Oncology* 11(1), 35–42. 10.37549/ARO1306

[CIT0035] Octavius, G.S. & Antonio, F., 2021, ‘Antecedents of intention to adopt mobile health (mHealth) application and its impact on intention to recommend: An evidence from Indonesian customers’, *International Journal of Telemedicine and Applications* 2021, 1–24. 10.1155/2021/6698627 PMC8105118 34012467

[CIT0036] Olausson, K., Holst Hansson, A., Zackrisson, B., Edvardsson, D., Östlund, U. & Nyholm, T., 2017, ‘Development and psychometric testing of an instrument to measure the patient’s experience of external radiotherapy: The Radiotherapy Experience Questionnaire (RTEQ)’, *Technical Innovations & Patient Support in Radiation Oncology* 3–4, 7–12. 10.1016/j.tipsro.2017.06.003 PMC7033812 32095560

[CIT0037] Park, S., Kim, H.-K., Choi, M. & Lee, M., 2021, ‘Factors affecting revisit intention for medical services at dental clinics’, *PLoS One* 16(5), e0250546. 10.1371/journal.pone.0250546 33945558 PMC8096099

[CIT0038] Purificacion, S., Brown, E., Anne-Davis, C. & French, J., 2016, ‘Patient engagement in radiation therapy: The development of guidelines for current Canadian practices’, *Healthcare Management Forum* 29(5), 187–195. 10.1177/0840470416647159 27576854

[CIT0039] Ringle, C.M. & Sarstedt, M., 2016, ‘Gain more insight from your PLS-SEM results: The importance-performance map analysis’, *Industrial Management & Data Systems* 116(9), 1865–1886. 10.1108/IMDS-10-2015-0449

[CIT0040] Sarstedt, M., Hair, J.F., Pick, M., Liengaard, B.D., Radomir, L. & Ringle, C.M., 2022, ‘Progress in partial least squares structural equation modelling use in marketing research in the last decade’, *Psychology & Marketing* 39(5), 1035–1064. 10.1002/mar.21640

[CIT0041] Shmueli, G., Sarstedt, M., Hair, J.F., Cheah, J.-H., Ting, H., Vaithilingam, S. & Ringle, C.M., 2019, ‘Predictive model assessment in PLS-SEM: Guidelines for using PLSpredict’, *European Journal of Marketing* 53(11), 2322–2347. 10.1108/EJM-02-2019-0189

[CIT0042] Stults, C.D., McCuistion, M.H., Frosch, D.L., Hung, D.Y., Cheng, P.H. & Tai-Seale, M., 2016, ‘Shared medical appointments: A promising innovation to improve patient engagement and ease the primary care provider shortage’, *Population Health Management* 19(1), 11–16. 10.1089/pop.2015.0008 26090793 PMC9238346

[CIT0043] Sung, H., Ferlay, J., Siegel, R.L., Laversanne, M., Soerjomataram, I., Jemal, A. & Bray, F., 2021, ‘Global Cancer Statistics 2020: GLOBOCAN Estimates of Incidence and Mortality Worldwide for 36 Cancers in 185 Countries’, *CA: A Cancer Journal for Clinicians* 71(3), 209–249. 10.3322/caac.2166 33538338

[CIT0044] Swain, S. & Kar, N.C., 2018, ‘Hospital service quality as an antecedent of patient satisfaction – A conceptual framework’, *International Journal of Pharmaceutical and Healthcare Marketing* 12(3), 251–269. 10.1108/IJPHM-06-2016-0028

[CIT0045] Tobiano, G., Jerofke-Owen, T. & Marshall, A.P., 2021, ‘Promoting patient engagement: A scoping review of actions that align with the interactive care model’, *Scandinavian Journal of Caring Sciences* 35(3), 722–741. 10.1111/scs.12914 33068042

[CIT0046] Wilcox, C.E., Mayer, A.R., Teshiba, T.M., Ling, J., Smith, B.W., Wilcox, G.L. et al., 2015, ‘The subjective experience of pain: An FMRI study of percept-related models and functional connectivity’, *Pain Medicine (Malden, Mass.)* 16(11), 2121–2133. 10.1111/pme.12785 25989475 PMC4653099

[CIT0047] Wolf, J.A., Niederhauser, V., Marshburn, D. & LaVela, S.L., 2014, ‘Defining patient experience’, *Patient Experience Journal* 1(1), 7–19. 10.35680/2372-0247.1004

[CIT0048] Wolf, J.A., Niederhauser, V., Marshburn, D. & LaVela, S.L., 2021, ‘Reexamining “defining patient experience”: The human experience in healthcare’, *Patient Experience Journal* 8(1), 16–29. 10.35680/2372-0247.1594

[CIT0049] Yan, X., Wang, J. & Chau, M., 2015, ‘Customer revisit intention to restaurants: Evidence from online reviews’, *Information Systems Frontiers* 17(3), 645–657. 10.1007/s10796-013-9446-5

[CIT0050] Yap, M.L., Zubizarreta, E., Bray, F., Ferlay, J. & Barton, M., 2016, ‘Global access to radiotherapy services: Have we made progress during the past decade?’, *Journal of Global Oncology* 2(4), 207–215. 10.1200/JGO.2015.001545 28717703 PMC5497622

